# Accurate chromatin marks peak calling with Omnipeak

**DOI:** 10.1093/nar/gkaf1454

**Published:** 2026-01-09

**Authors:** Oleg Shpynov, Maxim N Artyomov

**Affiliations:** JetBrains Research, Munich, Germany; Division of Immunobiology, Department of Pathology and Immunology, Washington University, School of Medicine, St. Louis, MO, United States; Division of Immunobiology, Department of Pathology and Immunology, Washington University, School of Medicine, St. Louis, MO, United States

## Abstract

Chromatin immunoprecipitation sequencing (ChIP-seq) is a widely used technique for identifying transcription-factor binding and histone modifications across the genome. Peaks in ChIP-seq data vary in length depending on the biological context, from narrow transcription factor binding sites to broad histone modification domains. However, commonly used peak-calling tools are tailored to the specific types of data and struggle to consistently handle various peak lengths, datasets of varying quality, and missing control tracks—common issues in comparative or meta-analyses. These limitations are addressed with Omnipeak, a universal unsupervised peak-calling algorithm based on a constrained three-state hidden Markov model. Omnipeak accurately models global genomic read coverage, capturing structure patterns in the data of all length scales and variable quality. We benchmarked Omnipeak versus eight different peak calling methods using over 550 public and 300 synthetic datasets, including conventional, ultra-low-input ChIP-seq, and ATAC-seq. Omnipeak produced consistent peaks across narrow, broad, and variable mark lengths, with the best agreement between replicates and robustness against noise and lack of control tracks. Together with a variety of supported input formats and peak calling capabilities within the genome browser, Omnipeak is well-positioned for processing various ChIP-seq and ATAC-seq datasets.

## Graphical Abstract

**Graphical Abstract:**
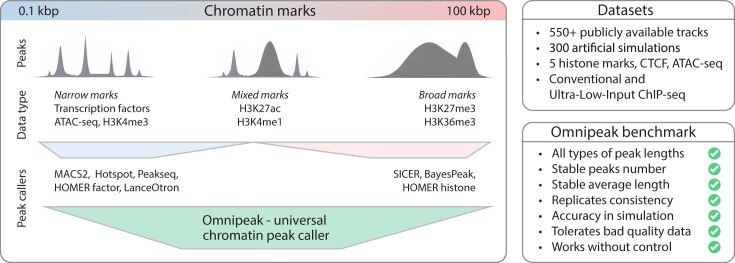

## Introduction

**Figure 1.**
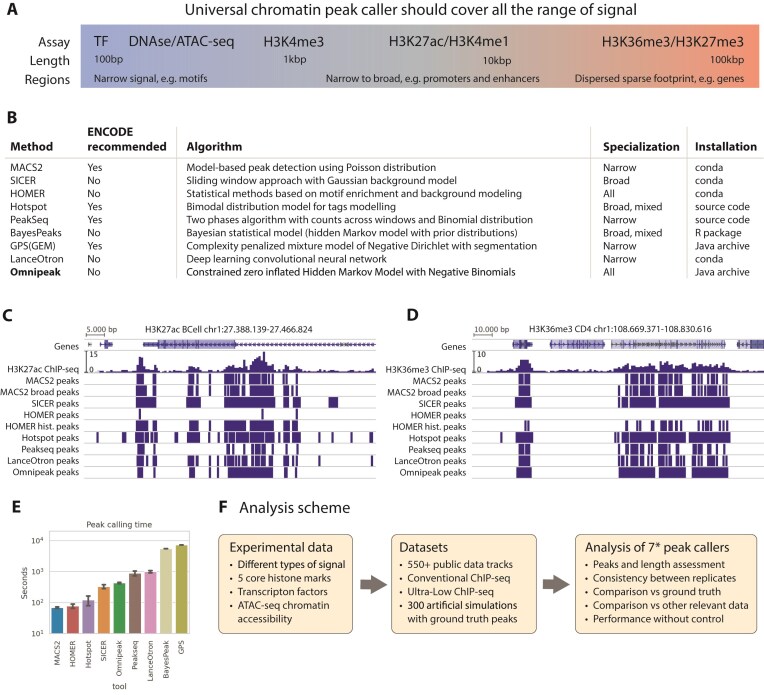
Peak calling methods landscape. (**A**) Overview of chromatin marks and typical lengths of open chromatin regions. (**B**) Summary of commonly used peak-calling methods, indicating ENCODE endorsement, underlying algorithm, histone mark specialization, and execution speed (for successfully processed cases). (**C**) Example of H3K27ac peak calling, illustrating how SICER, optimized for broad peaks, merges adjacent regions. (**D**) Example of H3K36me3 peak calling, showing fragmented results produced by MACS2 and LanceOtron, etc. (**E**) Average running time per experiment for each peak-calling algorithm on a limited dataset. (**F**) Schematic of the benchmarking workflow incorporating large public datasets with replicates, ultra-low-input datasets, and synthetically generated data. *Preliminary analysis included nine peak-calling algorithms, with MACS2 tested in narrow and broad modes, and HOMER in both default factor and histone modes. BayesPeak and GPS were excluded due to computational limitations, resulting in comparison of nine peak sets across >850 data tracks.

Chromatin immunoprecipitation followed by sequencing (ChIP-seq) [1] is widely used for mapping transcription-factor binding and histone modifications across the genome. This process involves high-throughput sequencing and subsequent peak calling—the computational task of identifying genomic regions with significant enrichment of sequencing reads, representing biologically relevant features such as transcription factor (TF) binding, histone modifications, or open chromatin, by distinguishing signal from background noise. TFs ChIP-seq produces the shortest peaks, a few hundred base pairs in length, as confirmed by the ChIP-Exo protocol [2]. The typical peak length distribution for a particular histone modification remains consistent [3] across diverse cell lines and datasets, and the ENCODE [4] consortium categorizes most histone marks as narrow or broad. For instance, narrow histone marks like H3K4me3, associated with promoter regions [5], usually have a length near one kilobase pair. In contrast, broad marks, exemplified by H3K36me3 and connected with RNA transcription [6], can be identified across entire gene bodies, spanning hundreds of kilobase pairs (Fig. 1A). However, certain histone marks, including H3K27ac, associated with active regulation [7] or the enhancer-associated mark H3K4me1 [8], are not limited to narrow or broad peak length. Both these marks can be detected across regulatory elements of varying lengths, ranging from short promoter regions to broad super-enhancer regions, influencing peak lengths. Furthermore, recent reports highlight that even seemingly narrow marks like H3K4me3 include broad domains connected with essential genes for maintaining cell identity [9].

The typical length of a histone mark peaks significantly shapes the overall distribution of reads across the genome, varying from a narrow and concentrated to a more dispersed but less dense coverage. Unsurprisingly, numerous computational tools, or “peak callers,” have been developed to analyze specific ChIP-seq data. These tools differ significantly in their underlying algorithms, specialization toward specific chromatin marks, producing a fragmented landscape of methods optimized for narrow (e.g. TFs and DNAse/ATAC-seq motifs), mixed (e.g. promoters and enhancers marked by H3K27ac/H3K4me1), or broad signal profiles (e.g. dispersed footprints such as gene-associated H3K36me3/H3K27me3). Indeed, even the ENCODE project offers an extensive, curated list of recommended peak callers, each tailored to specific data types and signal characteristics.

One of the questions we would like to address in this work is the applicability of a variety of peak caller algorithms (Fig. 1B) to experiments of different typical lengths, quality, etc. The most commonly used methods for narrow marks are MACS2 (Model-based Analysis of ChIP-seq) [10] and HOMER (Hypergeometric Optimization of Motif EnRichment) [11], whereas SICER (Spatial Clustering for Identification of ChIP-Enriched Regions) [12] is used for broad marks. MACS2 is even incorporated into ENCODE’s computational pipelines for TFs and histone marks. It utilizes local coverage modeling and excels with narrow peaks but exhibits noticeably poorer performance on broad marks with low coverage density, confirmed by independent benchmarks [13]. MACS2 utilizes local coverage estimation and identifies peaks by modeling coverage using the Poisson distribution. Another widely used tool, HOMER, integrates statistical modeling based on hypergeometric distributions with motif enrichment analysis to call peaks. Primarily designed for detecting TF binding sites (narrow peaks), HOMER also features an additional chromatin operational mode, which performs well on mixed and broad signal types by adjusting statistical thresholds. While there’s no well-established standard tool for broad ChIP-seq, SICER is the most extensively used. SICER evaluates small, non-overlapping windows for enrichment against the global model and merges adjacent peaks located closer than a user-defined threshold distance. This approach empowers SICER to produce broad peaks, especially relevant for histone marks such as H3K36me3 or H3K27me3.

Additionally, we considered Hotspot [14], Peakseq [15], and GPS (Genome Positioning System) [16] as the tools explicitly recommended by ENCODE for ChIP-seq analysis. Hotspot applies a bimodal distribution model combined with tag-density modeling to identify regions enriched for TF binding sites. Specifically optimized for narrow peak profiles (such as TF footprints from DNase-seq and ATAC-seq data), Hotspot efficiently distinguishes localized binding events but is less suitable for broad histone marks. PeakSeq is a two-phase peak-calling algorithm that first identifies candidate regions by scanning windows across the genome and subsequently evaluates their statistical significance using a binomial distribution model. It is mainly designed for narrow peaks, such as those produced by TFs, and operates effectively in scenarios with clear and localized enrichment signals. GPS utilizes complexity-penalized mixture models of negative binomial distributions with genome segmentation to precisely locate TF binding events. This approach excels in identifying narrow peaks characteristic of TF binding but may underperform with broader signals due to its stringent modeling constraints.

Next, we considered a hidden Markov model (HMM) [17] based algorithm, BayesPeaks [18]. It implements a Bayesian statistical approach using an HMM framework with prior probability distributions to detect enriched regions. This method specializes in broad and mixed peak signals, such as histone modifications with diffuse enrichment profiles, integrating biological priors to enhance detection accuracy in noisy datasets. Lastly, LanceOtron [19] represents an advance in the deep-learning field—it uses a convolutional neural network (CNN) architecture specifically trained to predict peaks across diverse signal profiles. This algorithm represents a modern machine-learning approach, capable of processing narrow, broad, and mixed ChIP-seq signals by learning complex patterns directly from training datasets, thus providing adaptability and generalization across chromatin modifications.

These peak callers demonstrate the diverse computational approaches employed in analyzing chromatin immunoprecipitation data. Each is optimized for particular types of genomic signals and offers distinct advantages based on the nature of the experimental dataset. These are the only part of ChIP-seq analysis tools we were able to install and launch without runtime errors on a modern system. See the full list of considered methods in the Supplementary Table S1.

To illustrate the differences in the results produced by different methods, we first applied all the tools mentioned above to examples of narrow and broad histone marks, namely H3K27ac and H3K36me3. An example of a peak calling for the narrow-mixed H3K27ac histone mark (Fig. 1C) illustrates how tools specialized on narrow marks are able to capture distinct peaks, while HOMER and SICER’s approaches effectively join adjacent peaks into broad ones. Figure 1D demonstrates an example of peak calling for the broad H3K36me3 histone mark, showcasing the fragmented peak calling produced by narrow-specialized methods such as MACS2 or LanceOtron, contrasted against broader peak identification by SICER.

Despite the extensive availability of the peak calling methods, almost no single peak caller efficiently covers the entire spectrum of chromatin ChIP-seq signals, ranging from narrow TF regions (~100 bp) to broad histone marks such as H3K36me3 (∼100 kbp). Moreover, the growing interest in epigenetic regulation has led to the generation of a substantial amount of data, including numerous tracks that capture different aspects of epigenomic modifications and interactions. This surge in data highlights both the need and the opportunity for a universal chromatin peak calling algorithm, ready for meta-analyses inspired by those conducted for transcriptional data [20]. In this work, we introduce a novel universal peak-calling algorithm, Omnipeak, specifically designed to handle all types of ChIP-seq experiments. At first glance, Omnipeak was able to capture both narrow and broad domains in H3K27ac (Fig. 1C) and broad peaks in H3K36me3 (Fig. 1D).

To robustly assess its performance, we conducted extensive benchmarking on a large compendium of real and synthetic datasets. Before applying all these different tools on such a high scale, we estimated their computational performance on five different ChIP-seq tracks of five core histone modifications, with the results depicted in Fig. 1E. We measured the time necessary to obtain a peak file from a BAM alignment file, which in some cases required additional preprocessing, e.g. conversion to pileup BED format or the generation of a BigWig file [21] with 1-basepair coverage resolution. According to this benchmark, all the tools can be separated into four different classes: fast—MACS2, HOMER, Hotspot; medium—SICER, Omnipeak; slow—Peakseq, LanceOtron; extra slow—BayesPeak, GPS. In the comprehensive analysis, we decided to proceed with all except the extra slow group, covering seven different peak calling methods, with MACS2 profiled in two different modes—narrow and broad—and HOMER factor (default) and histone modes.

Our evaluation scheme (Fig. 1F) provides a detailed comparison of our novel tool against existing methods, evaluating performance metrics such as peak and length accuracy, consistency across replicates, comparative accuracy versus ground truth, and performance without control samples. We benchmarked Omnipeak using over 550 public and 300 fully synthetic datasets, including conventional ChIP-seq, ultra-low-input data, and fully artificial datasets with defined ground-truth peaks. Omnipeak produced consistent peaks across narrow, broad, and variable mark lengths, with the best agreement between replicates and robustness against noise and lack of control tracks. Our results highlight Omnipeak’s versatility, accuracy, and efficiency across the entire range of chromatin signal profiles.

We describe this approach in detail in the following sections.

## Materials and methods

### Omnipeak algorithm

To address the lack of a universal chromatin peak calling solution, we introduce Omnipeak—an unsupervised three-state constrained HMM-based peak caller that leverages global and local signal characteristics. One primary advantage of an HMM model is its proficiency in detecting global patterns and trends in the entire dataset while abstaining from assuming any specific signal pattern. The constrained model learning procedure improves model quality of fit by keeping essential model features, such as noise level and signal-to-noise ratio, in data-dependent boundaries. Finally, the local peak shape adjustment enables Omnipeak to refine peak boundaries precisely, which is particularly beneficial for narrow signals.

**Figure 2.**
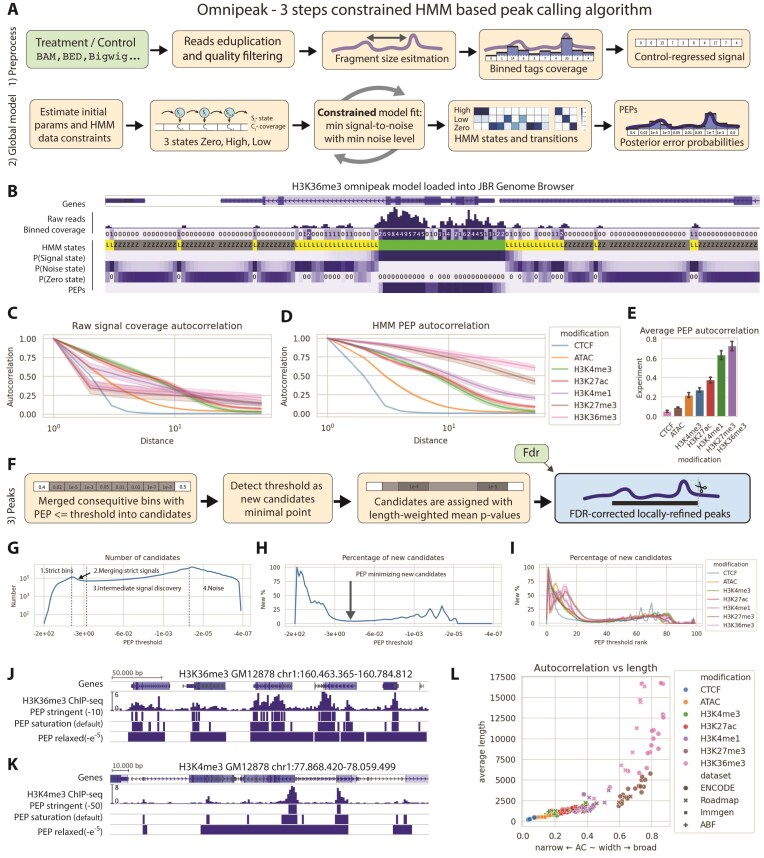
Omnipeak HMM model accurately captures structure of histone mark correctly. (**A**) Schematic representation of the OmniPeak workflow, including (1) data preprocessing and (2) constrained learning of the HMM. (**B**) Visualization of HMM states in the JBR Genome Browser using a real dataset. (**C**) Raw signal autocorrelation plotted by genomic bin shift. (**D**) Autocorrelation of HMM PEPs distinguishing characteristic patterns of different histone marks. (**E**) Average autocorrelation profiles across histone marks. (**F**) Peak definition from the HMM model (step 3 of the workflow). (**G**) Number of detected peak candidates as a function of the PEP threshold. (**H**) PEP saturation point illustrating the diminishing number of additional peak candidates. (**I**) Invariance of additional candidate detection with respect to the PEP threshold. (**J**) Example of H3K36me3 peak calling at varying PEP thresholds. (**K**) Example of H3K4me3 peak calling at varying PEP thresholds. (**L**) Average peak candidate length showing strong association with HMM PEP autocorrelation.

The algorithm comprises three key stages:

1. *Data Preprocessing*: The short reads coverage signal is binarized after standard read preprocessing and filtering, typically using bins of ∼50–200 bp. Subsequently, if a control track is provided, the signal is normalized to the control sample by minimizing the correlation between samples and controls (Fig. 2A top line; see the “Materials and methods” section for details).
2. *Genome-wide Constrained Model Fitting*: A statistical data model based on an HMM is fitted to the dataset during the second stage. The algorithm interprets read counts in the bins as a sequence of numerical observations falling into one of three HMM states: zero, noise, and signal, with noise and signal represented by negative binomial distributions. The choice of three states is grounded in the understanding that extensive genomic regions have no coverage, and regions with coverage arise from either background noise or genuine signal. The model offers enhanced robustness compared to two-state models, explicitly separating no-signal and noise states (see the “Materials and methods” section). Hidden states and parameters are inferred using the expectation–maximization Baum–Welch algorithm [22], with additional constraints validation after each iteration. Finally, for each bin, a posterior error probability (PEP) [23] of a null hypothesis (assuming no signal) is computed (Fig. 2A bottom line). Additionally, model states and PEPs can be visualized directly in the JBR Genome Browser [24], as shown in Fig. 2B. JBR Genome Browser is a fast universal genome browser, developed in the group for massive-scale data visualization and analysis, namely for the ABF dataset. Moreover, peak calling with Omnipeak can be performed directly from the JBR Genome Browser graphical user interface (see Supplementary Fig. S4).
  Differences between ATAC-seq and various types of ChIP-seq are readily visible in genome browser visualizations and can also be quantified using raw signal autocorrelation [3] (Fig. 2C). Autocorrelation reveals the unique characteristics of the signal, but alone is insufficient for accurately predicting typical peak lengths. In contrast, the Omnipeak data model effectively captures diverse signal structures and distinguishes signals with different typical peak lengths, which is demonstrated in PEP autocorrelation (Fig. 2D). Moreover, the average value of PEP autocorrelation on the shown range of distances can serve as an efficient and unsupervised classifier for identifying the type of experiment, as shown in Fig. 2E.
3. *Model-Based Peak Computation*: In the final step, peaks are identified using PEP values and thresholds (Fig. 2F). Candidate peaks are determined by applying a threshold to bin-level PEPs and grouping adjacent bins that exceed the threshold to form candidate peaks.

The process of identifying an appropriate threshold leverages the statistical characteristics of peak distributions that apply universally across all genomic mark scales. Omnipeak adopts a consistent method for determining the optimal threshold across varying epigenetic modifications.

As the PEP threshold changes, the number of candidate peaks does not follow a strictly increasing pattern, as some candidates may merge when thresholds are relaxed (Fig. 2G).

The nature of candidate peaks shifts through these stages: (1) detection of highly confident peaks with strict thresholds, (2) merging of closely spaced confident peaks, (3) identification of intermediate peaks with lower significance, and (4) inclusion of residual low-level noise as continuous peaks. Examples of candidate peaks across thresholds are shown in Fig. 2J for broad H3K36me3 marks and Fig. 2K for narrow H3K4me3 marks. Stringent thresholds isolate the most reliable peaks, whereas relaxed thresholds increase the capture of noise.

Omnipeak determines the optimal threshold by identifying the saturation point, where adding new candidates slows significantly as the threshold varies (Fig. 2H). The concept of saturation threshold selection is consistent across all tested experiments (Fig. 2I). This ensures the prioritized identification of biologically relevant candidates while minimizing the inclusion of noise.

Once the threshold is finalized and candidate peaks are identified, Omnipeak calculates the *P*-values of individual candidates, adjusted for multiple hypothesis testing. Peak boundaries are then refined using local coverage data. Interestingly, the lengths of candidate peaks are strongly associated with global PEP autocorrelation, reflecting the model’s accuracy (Fig. 2L).

This three-stage approach combines the advantages of a global model with local coverage, utilizing minimal to no user-defined parameters, except for the false discovery rate.

## Results

### Omnipeak produces high-quality peaks for a broad range of ChIP-seq experiments

**Figure 3.**
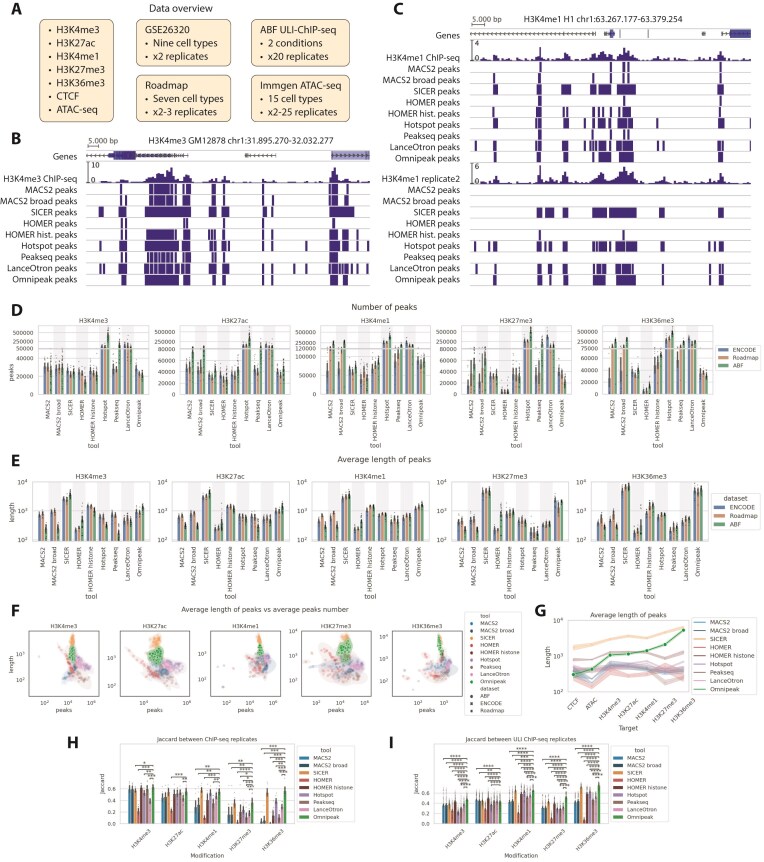
Omnipeak produces stable good-quality peaks for broad range of ChIP-seq experiments. (**A**) Overview of datasets and experimental data types used in the comparative analysis, including the number of samples and replicates. (**B**) Example of OmniPeak peak calling for H3K4me3, illustrating detection of broad domains. (**C**) Example of OmniPeak peak calling for H3K4me1 using replicate datasets. (**D**) Stable number of peaks detected by OmniPeak across multiple datasets. (**E**) Stable average peak length produced by OmniPeak across datasets. (**F**) Consistent peak number and average length across datasets demonstrating OmniPeak robustness. (**G**) Distribution of peak lengths showing that OmniPeak captures a broad range of genomic enrichment regions. (**H**) High replicate consistency of OmniPeak on conventional ChIP-seq datasets. (**I**) High replicate consistency of OmniPeak on ultra-low-input ChIP-seq datasets. Statistical significance was assessed using the two-sided Mann–Whitney–Wilcoxon test followed by Benjamini–Hochberg correction. Significance levels: * *P* ≤ .05, ** *P* ≤ .01, *** *P* ≤ 10^−3, ****^  *P* ≤ 10^−4^.

We conducted a comparative analysis of seven different peak calling algorithms, including Omnipeak, representing various computational approaches to peak identification, evaluating their performance on various histone marks, including narrow histone mark H3K4me3, various-length H3K27ac, H3K4me1, and broad H3K27me3 and H3K36me3, together with CTCF [25], a highly conserved TF that regulates genome organization (Fig. 3A). All the tools were used with recommended default parameters or recommended parameters in case of MACS2 broad peak calling mode. Histone marks assessment utilized three large publicly available datasets with replicates: the ENCODE (GSE26320 dataset [26]), a subset of the RoadmapEpigenomics datasets [27], and the ultra-low-input ChIP-seq [28] dataset with a high number of biological replicates, namely ABF [29]. Finally, we utilized ATAC-seq [30] chromatin accessibility data from the ImmGen project (GSE100738 dataset [31]) to assess peak calling quality when control is missing by experimental design. All datasets, ENCODE, Roadmap Epigenomics, and ABF, differ in total number of samples (Supplementary Fig. S1A), replicates (Supplementary Fig. S1B), and library depth (Supplementary Fig. S1C), with Roadmap Epigenomics averaging 21 million reads compared to ENCODE’s 8 million reads and >40 million reads of ABF. Such a discrepancy in library sizes provides additional obstacles for consistent peak calling [32].

Visual inspection is one of the most common ways of assessing raw data and peak calling quality [33], so Fig. 3B visualizes differences between tools for H3K4me3 of GM12878 cell line from ENCODE. Clearly, all the peak calling tools could be divided by their ability to detect narrow isolated peaks as well as broad domains. While narrow peaks tailored tools are perfectly suited for ultra-narrow TFs processing, they can make peak calling in broad domains of H3K4me3 and H3K27ac inefficient. On the other hand, tools with broad signal preference (e.g. SICER) tend to merge even clearly distinguishable individual peaks into one, which is suitable only for broad marks. In both cases, HOMER histone mode and Omnipeak were able to capture all types of peaks.

Replicated experiments generally allow for robust peak calling results by combining individual peaks from replicates. However, this becomes problematic when tools yield different or almost missing results for one of the replicates for the H3K4me1 mark of the H1 cell line from ENCODE, as depicted in Fig. 3C. In this case, only SICER, Hotspot, LanceOtron, and Omnipeak were able to detect comparable peaks in both replicates. We’ve prepared an online genome browser session for all the examples above; the links are available in Supplementary Table S3. However, an additional quantitative comparison is still required.

These patterns mentioned earlier are also visible on a global scale with full datasets. We examined the distribution of peak numbers and lengths to highlight global discrepancies in peak calling across three datasets. This analysis revealed differences not only between ultra-low-input and conventional ChIP-seq datasets but also within a single dataset type. For the narrow mark H3K4me3, most of the tools generated a consistent number of peaks across all three datasets, except Hotspot and Peakseq, with Hotspot, Peakseq, and LanceOtron producing significantly more peaks than the others (Fig. 3D). On H3K27ac, only SICER, HOMER, LanceOtron, and Omnipeak yielded a comparable number of peaks, with LanceOtron producing significantly more among them. Notably, narrow signal-tailored tools, such as MACS2 and HOMER for TFs (default), demonstrated worse results on broad histone marks. These patterns are observed among all five histone marks, with SICER, HOMER histone, and Omnipeak producing the least variable numbers.

Additionally, in some cases, algorithms produced peaks of different lengths for the same histone mark in different datasets (Fig. 3E), with MACS2 exhibiting the highest variation in all marks. This issue holds significant importance because MACS2 is widely used for ChIP-seq processing. Notably, the difference in hotspot length was observed for H3K4me3, while Peakseq showed variation for both H3K4me3 and H3K27ac, and HOMER (default) for H3K27me3. Remarkably, SICER constantly produced a stable number of broader peaks, while Omnipeak could produce almost stable numbers with consistent lengths corresponding to datasets.

The joint comparison of peak numbers with the average length of the peaks shows that Omnipeak yields stable results across all modifications and datasets, aligning results obtained from conventional and ultra-low-input ChIP-seq experiments (Fig. 3F).

A summary of the average peak length across datasets is shown in Fig. 3G, where the global patterns are clearly visible. Most algorithms produced peaks of quite a limited range, even for broad marks, specifically producing fragmented peaks on broad marks. SICER and HOMER produced a wider range of lengths, while only Omnipeak was able to capture the full range of lengths from narrowest TF binding sites to spacious H3K36me3.

Consistency between replicates is another important metric to consider when assessing peak calling quality. The Jaccard index was used to quantitatively estimate consistency, computed as the fraction of the intersection to the union lengths of two peak sets. We separately assessed consistency on conventional and highly variable ultra-low-input ChIP-seq experiments (Fig. 3H and I) to emphasize the importance of a novel universal tool. Most of the tools, excluding HOMER default and LanceOtron, generally produced consistent peaks for narrow histone marks on ChIP-seq and worse results for all types of ultra-low-input experiments. Broad marks, including H3K27me3 and H3K36me3, caused the most trouble in producing consistent results. In all cases where statistical significance is reported, it is determined using the two-sided Mann–Whitney–Wilcoxon test, followed by the Benjamini–Hochberg correction. Only significant results are shown, with the following notation: *: *P* ≤ .05, **: *P* ≤ .01, ***: *P* ≤ 10^−3^ , and ^****^: *P* ≤ 10^−4^ . In almost all cases, Omnipeak either has results comparable to the best or statistically better results. We provided the summary information based on the ranks of the results in the Supplementary Fig. S5.

The systematic application of the peak calling algorithm to a large number of publicly available experiments already highlights major behavior patterns and suggests the proper analysis tools, but additional comparison, either with biological signals of different types or on synthetic data, brings more insights.

### Benchmarking approaches

Several methods exist to evaluate the performance of peak-calling algorithms [34], including analyzing peak features (e.g. count, length, and replicate consistency [35]), comparing results to independent biological signals [36], or simulating reads with known ground truth [37]; see the full list in the Supplementary Table S4. In this work, we applied all these approaches to properly benchmark Omnipeak.

We analyzed publicly available ChIP-seq and ultra-low-input tracks, evaluating peak metrics, replicate consistency, and correlations with RNA-seq data for active gene transcription [36]. Using synthetic data generated with the Chips toolkit [37], we tested Omnipeak performance under varying noise levels with known ground truth and evaluated its robustness with and without control tracks. Finally, we assessed Omnipeak on the ATAC-seq ImmGen dataset, designed without control experiments.

### Omnipeak consistently matches narrow and broad marks associated with transcription

**Figure 4.**
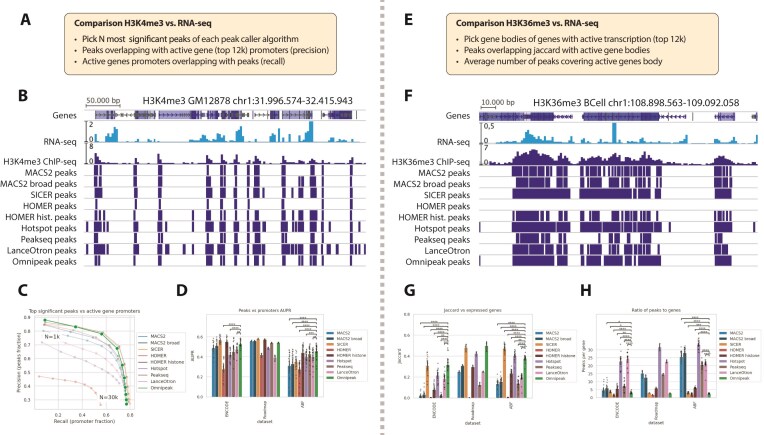
Omnipeak matches broad and narrow marks associated with transcription. (**A**) Schematic of the benchmarking strategy for H3K4me3 peaks against transcriptional RNA-seq data. (**B**) Example of H3K4me3 peak calling compared with RNA-seq read coverage visualization. (**C**) Representative precision–sensitivity plot for the H3K4me3 versus RNA-seq benchmark using ENCODE K562 cell line replicate 1. (**D**) Area under the curve (AUC) summary of precision–sensitivity performance across all datasets for different peak-calling tools. (**E**) Benchmarking scheme for H3K36me3 peaks against transcriptional RNA-seq data. (**F**) Example of H3K36me3 peak calling compared with RNA-seq read coverage. (**G**) Jaccard index quantifying the overlap between actively expressed genes (top 12k) and detected peaks. (**H**) Ratio of peaks to genes comparing expressed gene body regions (top 12k) with identified peaks.

RNA-seq experiments were utilized for accurate gene expression quantification, detection of actively transcribed genes, and comparison of H3K4me3 and H3K36me3 peaks with promoters and gene bodies of expressed genes. While the presence of H3K4me3 in promoter regions or H3K36me3 in gene bodies does not conclusively indicate active transcription, genes with a high signal in respective areas are more likely to be involved in transcription. We conducted an experiment (Fig. 4A) inspired by a previously published benchmark [36] to assess the consistency between peak signal confidence and transcription. By selecting the most significant *N* = 1000, 2000, …,15 000 peaks and overlapping them with the promoter regions or bodies of active genes, we computed the fractions of active gene regions overlapped by peaks, assessing the precision and sensitivity of peak calling methods.

Typical peak calling results for the H3K4me3 mark in the GM12878 cell line (ENCODE) are shown in Fig. 4B. The panel includes read coverage from the associated RNA-seq experiment, the H3K4me3 ChIP-seq experiment, and peaks produced by various algorithms. For H3K4me3, unsurprisingly, HOMER default and MACS2 produced the shortest peaks, and Omnipeak was close to MACS2 broad. At the same time, SICER identified the longest peaks (Fig. 4B). Overlapping active gene promoters with the most significant H3K4me3 peaks allowed us to evaluate the specificity and sensitivity of the methods, whose individual outcome is shown in Fig. 4C. HOMER histone, SICER, and Omnipeak yield the best results due to the best overlap with transcription start sites of the actively transcribed genes. The summary AUC for all ENCODE, Roadmap Epigenomics, and ABF datasets is shown in Fig. 4D. Omnipeak, together with SICER and HOMER histone, exhibits the most precise and sensitive results, surpassing other methods.

H3K36me3 is linked to transcriptional activity and associated with the genomic positions of expressed gene bodies, making it an appropriate metric for evaluating algorithm accuracies, so we compared the peak calling results with gene bodies of actively transcribed genes (Fig. 4E). An example of peaks for BCell (Roadmap Epigenomics) is shown in Fig. 4F. Most of the tools produced highly fragmented results, while only SICER, HOMER, Peakseq, and Omnipeak identified broad peaks that reasonably cover gene bodies. A direct comparison of all H3K36me3 peaks with active gene bodies (Fig. 4G) reveals that Omnipeak, SICER, and Hotspot peaks exhibit the best concordance with genes, with Omnipeak as the second best in one of the datasets. On average, only Omnipeak, SICER, and HOMER default yield under five peaks per single active gene body (see Fig. 4H), with Hotspot and LanceOtron producing >20 peaks on average. Interestingly, that MACS2 behavior was the most variable across datasets.

In all cases, Omnipeak showcased among the best performance in detecting peaks associated with actively transcribed genes for individual cell lines and the entire datasets. These results highlight Omnipeak’s capabilities to consistently produce biologically meaningful peak calling results.

### Omnipeak produces robust results in synthetic datasets of various quality

While we could assess the algorithms’ performance based on the characteristics of the detected peaks and their consistency with an independent RNA-seq dataset, we did not have a ground-truth reference for direct comparison. To address this limitation, we employed model-based ChIP-seq read simulations using Chips [37]. Chips can learn from real data and peaks to generate artificial reads for specified reference peaks. This approach considers various aspects of the ChIP-seq experimental process, including fragment size, polymerase chain reaction (PCR) amplification, library size, the fraction of reads within peaks, and more. The exact step-by-step procedure of simulation is provided at https://github.com/JetBrains-Research/peak-callers-analysis/blob/master/chips/Simulation.md.

**Figure 5.**
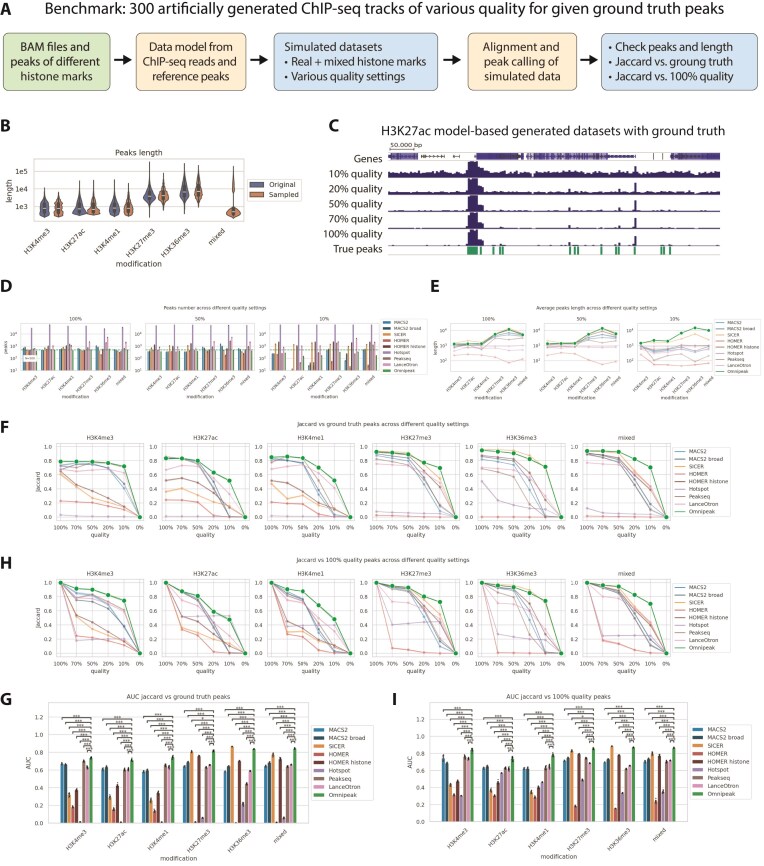
Omnipeak produces robust results in synthetic datasets of various quality. (**A**) Schematic of the benchmarking framework using Chips model–based artificial ChIP-seq experiments of varying quality with predefined ground-truth peaks. (**B**) Length distributions of original and sampled ground-truth peaks, including mixed tracks combining both narrow and broad peak types. (**C**) Example of H3K27ac Chips-generated datasets illustrating varying data quality. (**D**) Number of detected peaks across simulated datasets of different quality levels. (**E**) Average peak length in simulated datasets under varying quality conditions. (**F**) Jaccard index comparing peak-calling results with ground-truth peaks across quality levels. (**G**) AUC summary of Jaccard index performance relative to ground-truth peaks. (**H**) Jaccard index between peaks detected in 100% quality simulations and those in deteriorated-quality conditions. (**I**) AUC summary of Jaccard index values relative to the 100% quality benchmark.

As previously demonstrated, library size and data quality significantly influence peak calling results [32], a crucial consideration in ultra-low-input ChIP-seq experiments, where signal-to-noise is substantially lower than in conventional ChIP-seq protocols. To estimate the sole impact of data quality on peak calling results and mitigate the effects of library size and quality on the results, we systematically generated tracks with a fixed library size and various signal-to-noise ratios. We used Chips to simulate multiple (*N* = 300) raw read tracks of different quality—100%, 70%, 50%, 20%, and 10% (Fig. 5A). Notably, Chips produces tracks almost without any background noise on 100% quality datasets. In contrast, an average real-life dataset resembles 50%–10% quality settings of this simulation approach. The lengths of the original and reference ground truth peaks used for synthetic dataset generation are shown in Fig. 5B. Additionally, we created a mixed dataset by combining peaks from the narrow histone mark H3K4me3 and the broad histone mark H3K36me3. Examples of generated ground truth peaks and Chips-generated tracks of various quality can be seen in Fig. 5C.

Initially, we analyzed the number of peaks detected by all the algorithms with respect to the generated signal quality, as shown in Fig. 5D. Even though most algorithms identified ~500 peaks in the ground truth set, Hotspot systematically yields enormous peaks in all setups. Unsurprisingly, the highest variance between tools is visible on the noisiest tracks with quality = 10%.

Additionally, we analyzed the peak lengths produced by the tools in different setups (see Fig. 5E). Previously, we showed the differences in peak length captured by different tools—here, we could confirm our findings in a controlled simulated setup. Dataset quality has a significant impact not only on the number of peaks but also on the average length—most tools, except SICER and Omnipeak, fail to detect broad peaks under the most low-quality conditions.

Next, we compared the resulting peaks with ground truth using the Jaccard index, which is the length of the intersection divided by the length of the union of peaks. Performance per mark is illustrated in Fig. 5F, where the Jaccard score is presented for various quality conditions, and the summarized AUC is shown in Fig. 5G. AUC statistics demonstrated excellent Omnipeak performance in accurate peak calling with known ground truth, with Omnipeak, MACS2, Peakseq, and LanceOtron performing well on narrow marks, and Omnipeak, SICER, and HOMER histone on broad marks. Afterward, we compared peaks from 100% quality with lower-quality tracks (Fig. 5H). Summarized AUC confirmed Omnipeak results tolerance to data quality (Fig. 5I).

These experiments demonstrated Omnipeak’s highest tolerance for poor data quality. Notably, the artificial simulation highlighted that most of the algorithms were tailored to specific data patterns (e.g. HOMER histone and SICER demonstrated modest performance on narrow marks and good performance on broad marks), while Omnipeak demonstrated stable best performance even on truly mixed-length datasets.

### Omnipeak produces consistent high-quality results with and without a control track

Current knowledge suggests that the control track plays an important role in ChIP-seq analysis. It enables the high-confidence identification of true protein–DNA binding events while minimizing false positives introduced by background noise, technical biases, and open chromatin features.

Unfortunately, control tracks are typically unavailable in automated meta-analyses. Moreover, it was previously demonstrated that the absence of a control track can significantly impact results [38], so we explored the effect of the absence of a control track on the resulting peaks.

**Figure 6.**
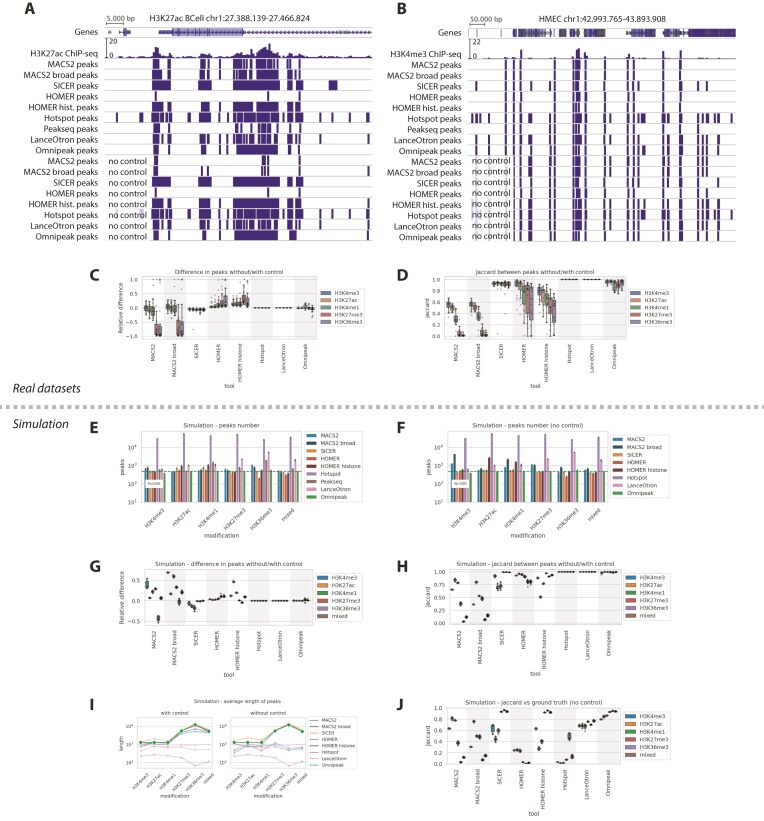
Omnipeak produces high-quality consistent results with and without a control track. (**A**) Example of H3K27ac peak calling performed with and without control data. (**B**) Example of H3K4me3 peak calling performed with and without control data. (**C**) Relative difference in the number of peaks detected without versus with control. The difference is defined as the fraction of differential peaks between the two conditions relative to the union of all detected peaks. (**D**) Jaccard similarity index between peaks identified with and without control data. (**E**) Number of peaks in the synthetic ground-truth dataset generated by the Chips toolkit (with control). (**F**) Number of peaks detected in the corresponding simulated dataset without control. (**G**) Relative difference in the number of peaks detected with versus without control across simulated datasets. (**H**) Jaccard index comparing peak-calling results with and without control in simulated datasets. (**I**) Length of peaks in simulation with and without control. (**J**) Jaccard index comparing peaks called without control against ground-truth peaks in simulations.

We performed the peak calling process without a control sample for all tools, except Peakseq, which requires the presence of a control track. Figure 6A illustrates peak calling results with and without control tracks for H3K27ac on all tested algorithms. In this example, MACS2 yields substantially fewer peaks when the control track is missing, especially in broad signal regions. Other tools produced more consistent results. In contrast, on a narrow mark, H3K4me3 MACS2 and HOMER in both modes produced more peaks than peak calling with control on broad marks (see Fig. 6B). SICER, HOMER, and Omnipeak are slightly affected by missing control. Surprisingly, LanceOtron and Hotspot visually demonstrated an absolute lack of dependency on control track presence, not using control information at all, which generally leads to inaccurate peak calling.

Next, we systematically compared the peak numbers produced without control to those with control. We calculated the relative difference as a ratio of the number of differential peaks to the number of peaks in overlap. The summary of the quantified relative difference of peak numbers is shown in Fig. 6C and D. MACS2 demonstrates high discrepancy in all cases, with both extra and missing peaks on H3K4me3, H3K27ac, and H3K4me1 marks, with a clear negative trend in broad marks H3K27me3 and H3K36me3. HOMER tends to produce slightly more peaks without control, especially on broad marks. Hotspot and LanceOtron were the least sensitive to control presence.

To ensure the consistency of the results, we further evaluated the similarity between peaks with and without control with the Jaccard index. Figure 6D illustrates that Omnipeak and SICER results exhibited the highest similarity after control-tolerant Hotspot and LanceOtron, exceeding 0.8, regardless of the presence of control. MACS2 presented more modest results, even for narrow histone marks (ranging from 0.4 to 0.7). HOMER results were consistently worse on longer marks in both factor and histone modes.

Additionally, we reproduced a similar analysis in well-controlled simulated conditions. We utilized the reference ground truth peaks and corresponding datasets previously generated by the Chips simulation toolkit. This time, we focused only on the highest-quality simulation results to eliminate quality issues in peak calling. Figure 6E and F illustrate the number of peaks identified by different tools with and without control. As previously, we computed the relative difference in the number of peaks together with the Jaccard similarity, as shown in Fig. 6G and H. MACS2 produced more peaks in H3K4me3 and H3K4me1 without a control track. HOMER histone reported more peaks on H3K27ac and H3K4me1 without a control, and the numbers for other algorithms were nearly identical. As in real datasets, Hotspot and LanceOtron reported absolute tolerance to the control track presence; MACS2 was the most variable, followed by HOMER, SICER, and Omnipeak. Jaccard comparison highlighted that Omnipeak was able to report the most similar peaks in both conditions. Moreover, we also compared the length of peaks produced by various algorithms with and without control tracks (Fig. 6I)—again, only SICER and Omnipeak detected long peaks in broad marks without a control track. Jaccard similarity versus reference peaks additionally confirms our findings (Fig. 6J)—Omnipeak produced the most stable results across all the tools. All the above suggest that one should practically use Omnipeak for the experiment with the missing control track.

### Omnipeak for ATAC-seq analysis

One scenario where the ability to reliably call peaks in the absence of control tracks is critical is ATAC-seq data, one of the most common methods for assessing epigenetic accessibility of the genome. ATAC-seq is a powerful technique for studying chromatin accessibility and is practically applicable when studying chromatin dynamics during different cellular processes, development, or disease states. ATAC-seq typically does not imply a control profiling experiment because the Tn5 transposase (an enzyme used in ATAC-seq) preferentially inserts into regions not occupied by histones or tightly packed nucleosomes, making the experimental signal inherently represent only accessible chromatin regions. The increasing use of single-cell ATAC-seq [39], which reveals the unique chromatin states of different individual cells or subpopulations, further highlights the importance of selecting an appropriate analysis tool because major computational single-cell ATAC-seq pipelines still contain bulk signal peak calling steps [40]. We hypothesized that Omnipeak’s effectiveness in analyzing ChIP-seq data without control experiments makes it a potentially very good tool for ATAC-seq.

We applied all the tools (except Peakseq) to the ImmGen ATAC-seq dataset, a part of the Immunological Genome Project that specifically provides data on chromatin accessibility across a wide range of immune cell types in mice. The dataset comprises a large number of various immune cell types. As a ground truth, we compared ATAC-seq peaks against DNase I hypersensitive sites (DHSs) [41], which are regions of chromatin that are accessible and “open” to the enzyme DNase I, which preferentially cuts DNA where nucleosomes are absent or disrupted. Both DHS and ATAC-seq identify open chromatin, so we used mm10 DHS representative sites track to compare results provided by both independent experiment types.

**Figure 7.**
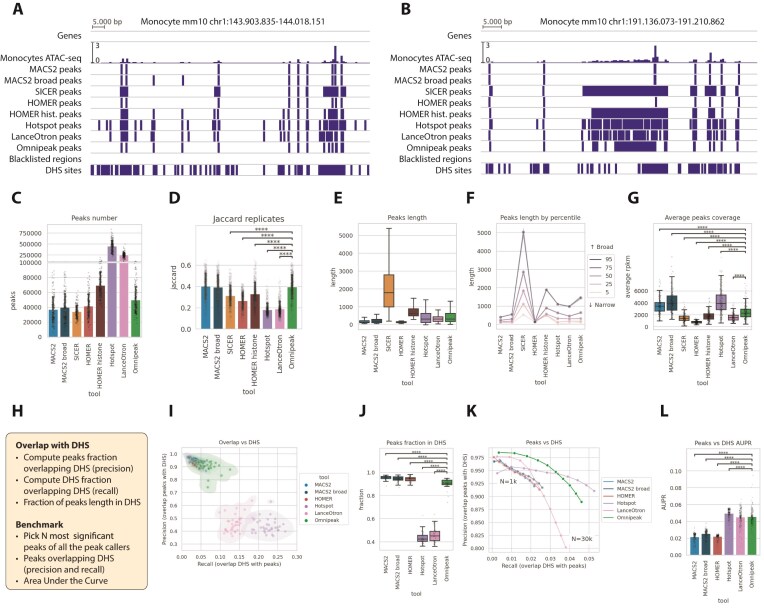
Omnipeak produces high-quality results for ATAC-seq consistent with DNase. (**A**) Example of peak-calling results from a representative ImmGen ATAC-seq experiment. (**B**) Example of peak calling on an ImmGen ATAC-seq signal cluster (outside of blacklisted genomic regions). (**C**) Number of peaks detected by different peak-calling tools across the entire ImmGen dataset. (**D**) Jaccard similarity index between replicate experiments within the ImmGen dataset. (**E**) Distribution of ATAC-seq peak lengths across peak-calling tools. (**F**) Detailed composition of peak lengths by percentiles (5th, 25th, 50th, 75th, and 95th) for each tool. (**G**) Average read coverage (RPKM) for the top 1000 most significant peaks. (**H**) Schematic of the benchmarking workflow comparing detected peaks with DNase I hypersensitive sites (DHS). (**I**) Overlap between peaks and DHS regions for all peak-calling tools except SICER and HOMER (histone mode). (**J**) Fraction of total peak length overlapping DHS regions. (**K**) Precision–recall curves for the N most significant peaks overlapping DHS regions. (**L**) AUC summary for all tracks from ImmGen dataset.

We started by visualizing peaks produced by different tools (Fig. 7A). SICER and HOMER histone tend to merge adjacent individual peaks, while others yield more similar peaks (see Fig. 7A). Interestingly, Omnipeak can identify ATAC-seq peaks not detectable by MACS2, but also captured by other tools, as shown in Fig. 7B. Most of the peaks agree with DNase hypersensitive sites, shown as a separate track on visualizations.

Dataset-wide, Omnipeak produced more peaks than MACS2 (50k versus 35k for MACS2 and 40k for MACS2 broad), HOMER (default), and SICER (Fig. 7C). HOMER histone was able to identify 60k+ peaks, with LanceOtron and Hotspot producing >250k, which perfectly confirms the LanceOtron authors’ information about LanceOtron’s high specificity.

Replicates consistency analysis highlighted the fact that more peaks don’t always mean better results. The abundance of peaks produced by Hotspot, LanceOtron, and even HOMER leads to lower inter-replicate consistency, as depicted in Fig. 7D. Omnipeak was able to capture larger number of peaks than MACS2, still ensuring the best-presented consistency among tools.

Omnipeak showed its capability in detection not only of separate peaks, but also clusters of ATAC-seq, so we analyzed the lengths of produced peaks, which is shown in Fig. 7E. Since an average length may be highly biased by the longest elements, we compared the typical peak length of the tools in a percentile manner (Fig. 7F)—the higher percentile value the longer peaks. Up to the 50th percentile, which corresponds to individual peaks, Omnipeak identified peaks comparable with MACS2, Hotspot, LanceOtron, and HOMER factor, but shorter than HOMER histone and significantly shorter than SICER. Peaks in high percentiles by lengths correspond to the clusters of ATAC-seq. Notably, HOMER factor (default) produced the less variable length of peaks.

Next, we analyzed the coverage density (reads per kilobase pair per million) of the top thousand most significant peaks reported by the tools, as shown in Fig. 7G. Omnipeak’s most significant peaks yield a read density less than MACS2 and Hotspot, but statistically better than other tools.

After, we compared the produced peak with DHS hypersensitive sites (see Fig. 7H–L). First, we excluded SICER and HOMER histone from the analysis, since they don’t capture short ATAC-seq peaks, and compared the overall overlap of peaks with DHS (Fig. 7I), which indicated that Omnipeak recall (fraction of DHS sites covered by peaks) and precision (fraction of peaks overlapping DHS) are close to MACS2 and HOMER default, with LanceOtron and Hotspot producing more sensitive results but significantly worse precision. Also, we assessed the peak length fraction, which is covered by DHS hypersensitive sites (Fig. 7J). It shows that even though the Omnipeak fraction of peaks covered by DHS was statistically lower than those of MACS2 and HOMER, it was only by a small margin, and still significantly bigger than Hotspot and LanceOtron.

To assess the overall performance of ATAC-seq peak calling compared to DHS, we employed a familiar procedure to compute AUC-based overlap metrics. We selected the top N significant ATAC-seq peaks and overlapped them with DHS sites (Fig. 7K). The summary AUC values are presented in Fig. 7L. Hotspot showed the best performance, with LanceOtron and Omnipeak sharing the second-best performance, which was substantially higher than that of other tools.

This analysis additionally highlights Omnipeak’s ability to produce biologically meaningful peaks for ATAC-seq datasets and its potential applicability for single-cell ATAC-seq data, where the bulk peak calling step is common to most existing computational pipelines.

Finally, the Supplementary Fig. S5 contains all the comparisons of Jaccard and AUC performance presented as ranks, where the lower the rank is, the better the overall performance of the tool. It contains results for replicate consistency for chromatin marks and ATAC-seq, benchmarking results versus RNA-seq transcriptional data, evaluation in simulation with known ground truth, and performance without control tracks. In almost all these cases, Omnipeak produced either the best results or close to the top performers.

### Processing public datasets

ChIP-seq data from ENCODE, Roadmap Epigenomics, and Immgen were downloaded in fastq format. Data from ABF were downloaded in BED pileup format. Upon completion of the experiments, 120 histone mark tracks were processed for ENCODE, 56 histone marks for the Roadmap Epigenomics, and 192 for the ABF dataset, together with 9 CTFC tracks and 185 ATAC-seq datasets for the ImmGen project, summing to 562 publicly available datasets. Supplementary Table S2 contains comprehensive dataset details, including download information for each dataset. All datasets were uniformly processed either from raw reads (fastq) or from alignment (bam or bed.gz) files using an open-source snakemake-based pipeline [42], with standard quality control, alignment, visualization, and peak calling steps. ENCODE datasets were downloaded using sra-toolkit [43] according to SRR numbers. RNA-seq data were downloaded as precomputed quantified TMM counts produced by the RSEM method [44] in the ENCODE project computational pipeline for RNA-seq data. DHS representative sites for the mm10 genome were downloaded from the encoreproject.org portal. Classical human monocyte ChIP-seq data were downloaded from ENCODE. Blacklisted regions for hg38 and mm10 genomes were downloaded from the ENCODE blacklist GitHub [45]. Data from the ABF dataset were downloaded in bed.gz format and translated into BAM format using the bamToBed utility from bedtools [46]. Visualization of read coverage was computed with the bamCoverage utility from deepTools [47]. Coverage of specific genomic regions was computed using the pyBigWig library from deepTools, based on the count-per-million normalized bigwig coverage files. Peak overlaps were computed with the help of bedtools and PyRanges [48]. We provide the source code of all the analyses—see the “Code availability” section below.

### Omnipeak data preprocessing

Omnipeak supports aligned reads in BAM, SAM, CRAM, BED, BED.gz, and BigWig file formats. There are no restrictions on supported genome assemblies—the only requirement is a file with chromosome sizes. Omnipeak supports both single-end and paired-end sequencing libraries. First, alignment files are filtered for redundant duplicate reads to avoid possible PCR duplication artifacts. Library fragment size is estimated by the cross-correlation for single-end libraries [49] or computed based on paired-read positions for paired-end cases. Tags from sense and antisense strands are shifted toward the fragment centers, significantly improving the narrow peak patterns' spatial resolution. Then, the total coverage profile is computed across small, non-overlapping bins. The last step of the preprocessing part is control correction, which is performed by introducing a beta value [3], minimizing the correlation between the control-corrected signal and the control track. Beta value is chosen from the range 0 to 1, minimizing the correlation between control and signal minus control multiplied by beta.

### Omnipeak three-state HMM

We used three-state HMM to model and estimate ChIP-seq reads coverage in consecutive bins. Introducing a dedicated zero state significantly improved the quality of fit by covering massive genomic loci without any read signal. The second and third hidden states, referred to as noise and signal, mark bins carrying signal coverage that is either low or high and are based on a negative binomial distribution. We used the modified Baum–Welch algorithm [22] with constraints for model parameter fitting.

Model initialization and model validation are two critical aspects in fitting the statistical data model during the second phase of the Omnipeak algorithm. The accurate initial estimation of model parameters can optimize the fitting process and require fewer iterations to achieve model convergence. For non-zero states, parameters are initialized using the method of moments. Experimental data is used to estimate the expected signal-to-noise ratio and noise average level. The model takes around 10 iterations to reach saturation in quality fit, increasing the computational speed. After each algorithm iteration step, we validate constraints and adjust model state parameters to fulfill them if necessary. Constraints include a minimal signal-to-noise (SNR) ratio (ratio between signal and noise states means) and a minimal noise level. Experimentally, it was shown that the SNR threshold primarily affects narrow marks while keeping a minimal required noise level guards against too extensive peak calling in all histone marks. Constraints hyperparameters optimization was done for part of the datasets simultaneously (see Supplementary Fig. S2). It was previously reported that an additional validation step is required to ensure the model’s reliability—when applying HMM to ChIP-seq data, signal and noise states might eventually be swapped during the EM algorithm [50]. Omnipeak constraints check makes additional validation obsolete and guarantees that HMM can effectively detect noise. Finally, PEPs are computed for each bin.

### Omnipeak procedure for defining peaks from the model

Next, peaks are defined based on the model. Candidate peaks are computed based on thresholding PEPs. The number of candidates and average length are evaluated for each PEP threshold. The pivotal PEP points are detected from the candidates’ average length versus number plot (Supplementary Fig. S3). Importantly, using a log scale for PEP thresholding allowed the detection of pivotal points. The required PEP threshold is defined and computed as a saturation point of newly added candidates during threshold relaxation between pivotal points. Subsequently, bins passing the selected threshold are grouped into candidate peaks. The log *P*-value for each candidate peak is derived as a length-weighted average of the log *P*-values of subsequent blocks of the top 50% confident bins. These *P*-values are calculated using a local Poisson model compared to control reads and adjusted for multiple hypothesis testing using the Benjamini–Hochberg or Bonferroni [51] procedure. Finally, peak boundaries are refined using local coverage data. Peaks are saved to the user-provided path using the BED 6 + 3 file format.

### Snakemake peak calling pipeline

We developed a dedicated Snakemake-based pipeline for uniform ChIP-seq and ATAC-seq processing utilized in this work, simplifying the comparison of multiple datasets and meta-analyses. It streamlines a unified analysis from raw reads alignment to peak calling, integrating all the peak caller methods mentioned in this work. The pipeline is available on GitHub at https://github.com/JetBrains-Research/chipseq-smk-pipeline. All command line options for peak callers are provided in Supplementary Table S1, located in the dedicated Parameters tab. The source code, including all the steps to reproduce the results from this work, is also available on GitHub at https://github.com/JetBrains-Research/peak-callers-analysis.

Namely, we used the following command to launch the processing pipeline using Snakemake:

snakemake --printshellcmds -s <chipseq-smk-pipeline>/Snakefile all --cores all --use-conda --directory $(pwd) --config genome=hg38 start_with_bams=True macs2=True sicer=True omnipeak=True homer=True hotspot=True peakseq=True lanceotron=True,

where <chipseq-smk-pipeline> stays for the directory with the pipeline’s source code.

We used additional parameters for MACS2 broad, recommended in the official documentation:

macs2_mode=broad macs2_params=“--broad --broad-cutoff 0.1″ macs2_suffix=broad0.1,

and for HOMER histone:

homer_style=histone homer_suffix=regions.bed.

For the ImmGen dataset we added the following recommended bowtie2 options for ATAC-seq alignment files and Omnipeak:

bowtie2_params=“-X 2000 --dovetail” omnipeak_fragment=0.

### Simulation of artificial data with Chips

CD14 classical monocytes ChIP-seq tracks were used to perform peak calling by MACS2 and SICER. MACS2 peaks were used for H3K4me3, H3K27ac, and H3K4me1 histone modifications for Chips [37] to learn their models from raw alignment and reference peaks (Supplementary Table S2). SICER peak calling results were used as a reference for H3K27me3 and H3K36me3 histone marks. Due to technical limitations, we focused on 500 ground truth peaks and generated 1 million reads on chromosome 15 of the hg38 reference genome. Ground truth peaks were sampled independently for each run from reference peaks for histone mark, keeping the desired distance between peaks at least five kilobase pairs. When creating a genuine mix of datasets of narrow and broad peaks, we focused on 400 narrow and 100 broad peaks, as the latter have a higher typical length and significantly affect summary Jaccard metrics. All the code for model learning and simulations can be found on GitHub.

### Omnipeak integration with JBR genome browser

Omnipeak is tightly integrated with the JBR Genome Browser [24], and the user can perform peak calling directly within the browser in both unsupervised and semi-supervised modes. In the default unsupervised mode of Omnipeak, the user can directly upload BigWig or BAM files of ChIP-seq and optional control, select them, and launch peak calling from the popup menu. Afterward, the peaks file or the Omnipeak model is visualized for further visual inspection. See the example in Supplementary Fig. S4.

### Other chromatin profiling methods

CUT&Tag [52] (Cleavage Under Targets and Tagmentation) is an efficient and contemporary technique for profiling protein–DNA interactions and chromatin landscapes in cells, offering a faster and lower-input alternative to conventional ChIP-seq. To assess the performance of Omnipeak in processing CUT&Tag data, we analyzed H3K4me3 and H3K27me3 datasets from the GEO series GSE124557 [52]. Each dataset comprised two biological replicates, enabling both a comparison with conventional ChIP-seq data from the same cell line and chromatin marks, as well as an evaluation of replicate consistency. We used Omnipeak with default parameters for processing CUT&Tag read files in .bed.gz format. Representative CUT&Tag peaks identified by Omnipeak, along with corresponding ENCODE ChIP-seq peaks, are shown in Supplementary Fig. S6A. The Jaccard similarity analysis demonstrated high concordance between replicates and good agreement with ENCODE ChIP-seq results (Supplementary Fig. S6B and C). We used built-in Jaccard analysis feature available from the context menu right within the JBR Genome Browser.

## Discussion

Peak calling can be challenging due to inevitable differences between ChIP-seq or ATAC-seq datasets. These differences often arise from variations in cell types, tissue specificity, and environmental conditions, which affect TF binding and histone modifications readout. Additionally, technical factors like experimental design, sequencing depth, and biological replicates can influence the observed data, reflecting true biological variability and experimental inconsistencies. Peak calling area is highly saturated with various algorithms based on different principles and tailored to different experiment types. However, after processing many publicly available datasets, we observed limitations of major peak callers available to date, including inconsistencies in peak calling, the typical output lengths of the tools, which additionally confirms the need for a universal approach. The underlying algorithms don’t fully cover the whole length range of various histone modifications or ATAC-seq, further complicating meta-analyses. In contrast, Omnipeak reconsiders peak calling as a unified task, which shifts the paradigm of peak calling from a set of highly specialized tools. It addresses differences in datasets by modeling chromatin states probabilistically, with a constraint-based HMM-based approach as a good compromise between model complexity and computational efficiency, together with the unique unified peak calling procedure from model PEPs. The method also facilitates the study of biologically meaningful signal widths, including broad H3K4me3 domains linked to cell identity and age-related chromatin changes and the structural features of ATAC-seq signals. Traditional narrow-focused peak callers often miss or fragment these regions.

Omnipeak’s unified approach produces consistent, high-quality peak annotations that can serve as a foundation for advanced data-driven methods, including training large language models or CNNs for epigenomic research. For example, modern deep learning methods such as LanceOtron tend to favor narrow peaks, likely reflecting training on narrow-peak datasets, which could be improved by providing better training datasets.

Among the method’s limitations is the bin-based nature of the data analysis. While we implemented a signal-sharpening postprocessing step, Omnipeak is still limited in precisely detecting ultra-narrow signals close to the discretion bin size, e.g. precise nucleosome positioning in ATAC-seq experiments [53]. In single-cell ATAC-seq experiments, when performing bulk peak calling, reads representing accessible chromatin regions may vary across individual cells, which can lead to the merging of adjacent regions by Omnipeak. To address this issue, we implemented a dedicated --summits command-line option that enables Omnipeak not only to distinguish peaks from background noise but also to identify independent summits within broader peak regions. Another issue is not the best-in-class computational performance, even though we heavily rely on modern CPU scalar computation optimizations, which still leaves room for improvement.

## Supplementary Material

gkaf1454_Supplemental_Files

## Data Availability

Information about all the publicly available datasets used in this work is provided in the Supplementary Table S2.

## Code availability

Omnipeak is open-source software and is available for download at https://github.com/JetBrains-Research/omnipeak (https://zenodo.org/records/17803423). The source code for the technical ChIP-seq and ATAC-seq processing Snakemake pipeline is available at https://github.com/JetBrains-Research/chipseq-smk-pipeline (https://zenodo.org/records/17803444). The source code of the analysis described in this work is available at https://github.com/JetBrains-Research/peak-callers-analysis (https://zenodo.org/records/17799980).
