# Supplementary material for: Accurate chromatin marks peak calling with Omnipeak
Source: Nucleic Acids Res. 2026 Jan 9;54(1):gkaf1454. doi: 10.1093/nar/gkaf1454 (PMC12784980; doi:10.1093/nar/gkaf1454)
Supplement: gkaf1454_Supplemental_Files [file gkaf1454_supplemental_files.zip › 10_S3.pdf]

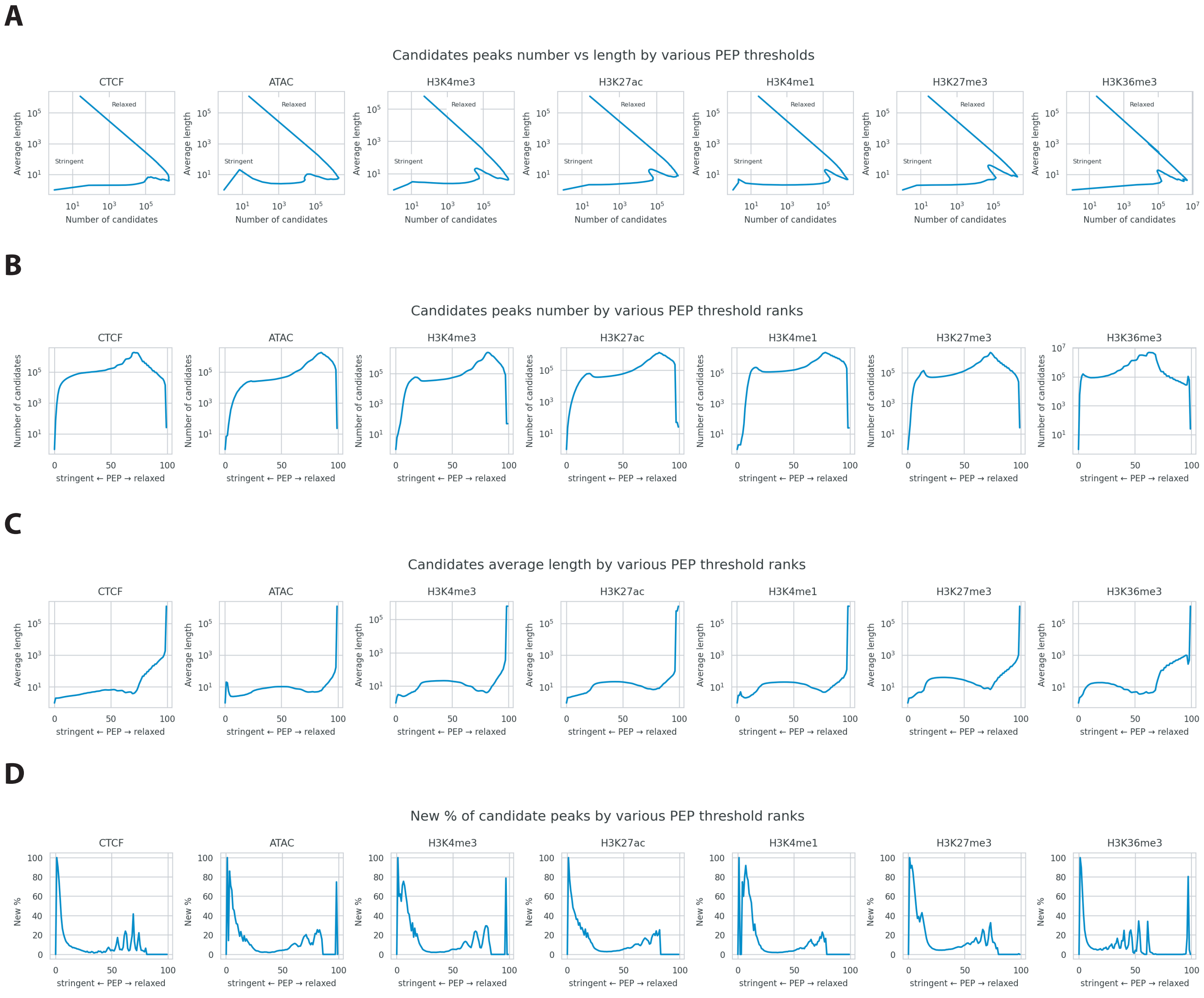

Figure S3 | Omnipeak candidate peaks patterns look similar across marks - a key to unified procedure.

A, Candidate peaks selection procedure involves analysis of both candidates number and average candidates length, during PEP threshold variation from the most stringest to the most relaxed settings.
Similar patterns present across all experiment types.

B, Similar patterns of number of candidate peaks vs various PEP theshold ranks across different experiments.
In all cases there is a steady growth, saturation and optionally small decline, growth
caused by yielding lots of unsignificant candidates and final merging for most relaxed PEP thresholds.

C, Candidates average length pattern is invariant across experiments, similar trajectory path as in number of peaks.

D, Visualization of novel candidates percentage while relaxing PEP threshold allows to detect saturation point.
