# Supplementary material for: Accurate chromatin marks peak calling with Omnipeak
Source: Nucleic Acids Res. 2026 Jan 9;54(1):gkaf1454. doi: 10.1093/nar/gkaf1454 (PMC12784980; doi:10.1093/nar/gkaf1454)
Supplement: gkaf1454_Supplemental_Files [file gkaf1454_supplemental_files.zip › 11_S4.pdf]

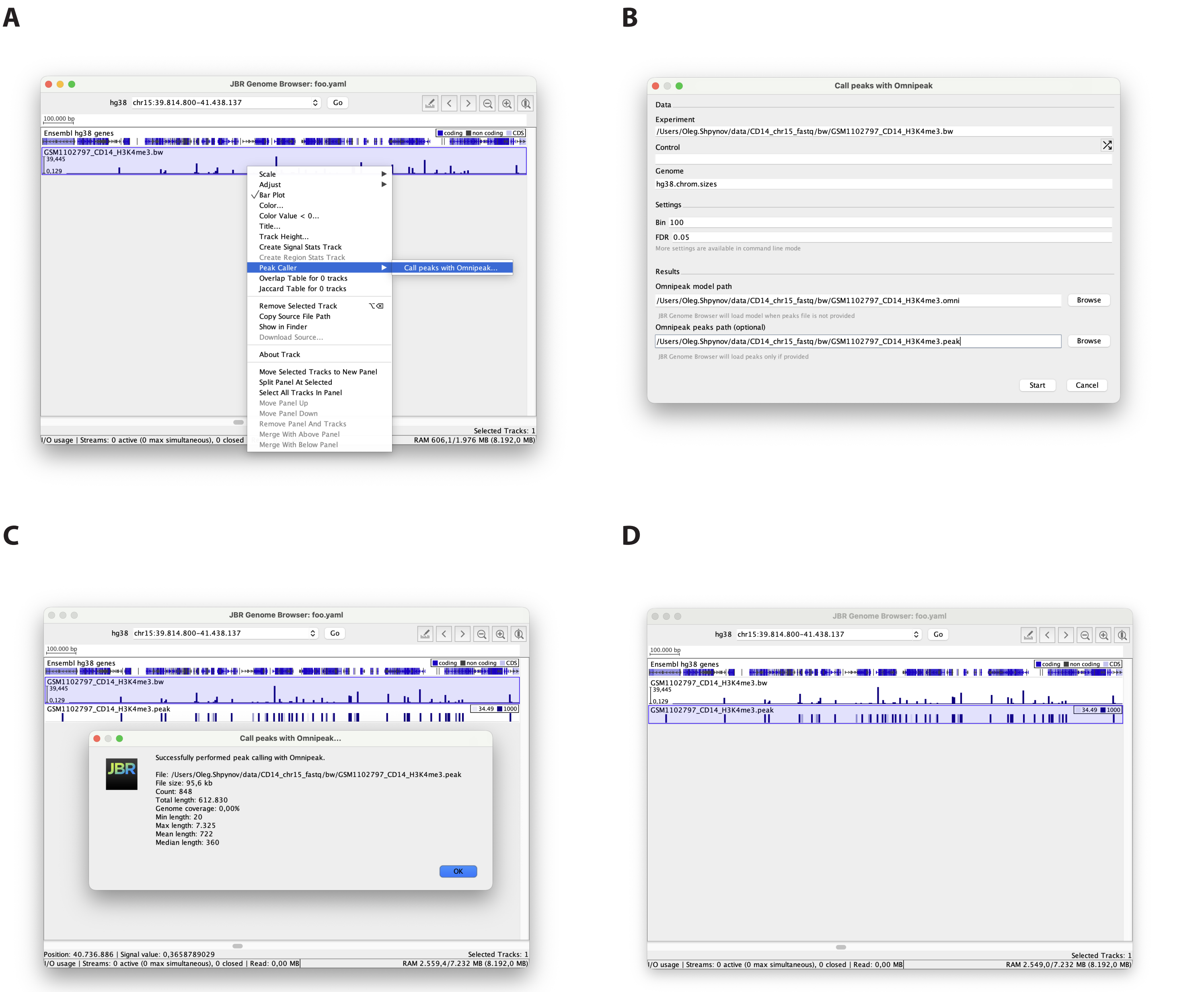

Figure S4 | Peak calling within JBR Genome Browser with Omnipeak.

A, User starts peak calling by selecting BigWig or BAM files and choosing *Peak Caller | Call peaks with OmniPeak* option from the JBR Genome Browser context menu.

B, Setting for peak calling including model and peaks location selection.

C, Summary information of the generated peaks following OmniPeak execution.

D, Visualization of the resulting peaks within the JBR Genome Browser user interface.
