# Supplementary material for: Accurate chromatin marks peak calling with Omnipeak
Source: Nucleic Acids Res. 2026 Jan 9;54(1):gkaf1454. doi: 10.1093/nar/gkaf1454 (PMC12784980; doi:10.1093/nar/gkaf1454)
Supplement: gkaf1454_Supplemental_Files [file gkaf1454_supplemental_files.zip › 12_S5.pdf]

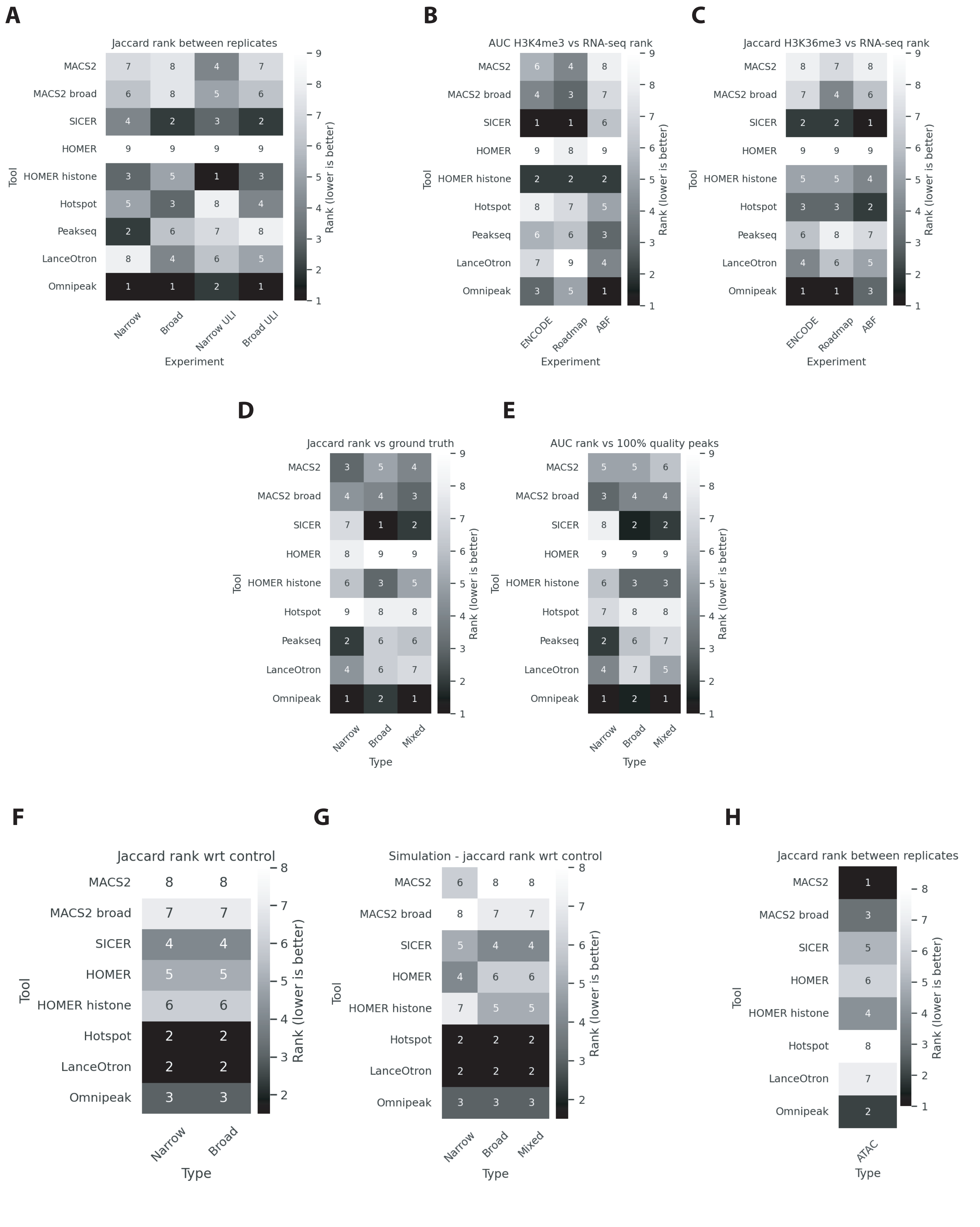

Figure S5 | Summary rank statistics of the benchmarks (the lower the better).

A, Ranks of the average rank of Jaccard between replicates.

B, Ranks of H3K4me3 most significant peaks versus RNA-seq actively transcribed genes.

C, Ranks of the H3K36me3 Jaccard overlap with active genes.

D, Ranks of the Jaccard AUC versus ground truth peaks in simulation with Chips.

E, Ranks of of the Jaccard AUC versus peaks obtained in 100% quality simulation.

F, Ranks of the Jaccard of peaks obtained with and without control tracks.

G, Ranks of the Jaccard of peaks obtained without control tracks vs ground truth peaks in simulation.

H, Ranks of the Jaccard between replicates in ATAC-seq dataset.
