# Supplementary material for: Accurate chromatin marks peak calling with Omnipeak
Source: Nucleic Acids Res. 2026 Jan 9;54(1):gkaf1454. doi: 10.1093/nar/gkaf1454 (PMC12784980; doi:10.1093/nar/gkaf1454)
Supplement: gkaf1454_Supplemental_Files [file gkaf1454_supplemental_files.zip › 13_S6.pdf]

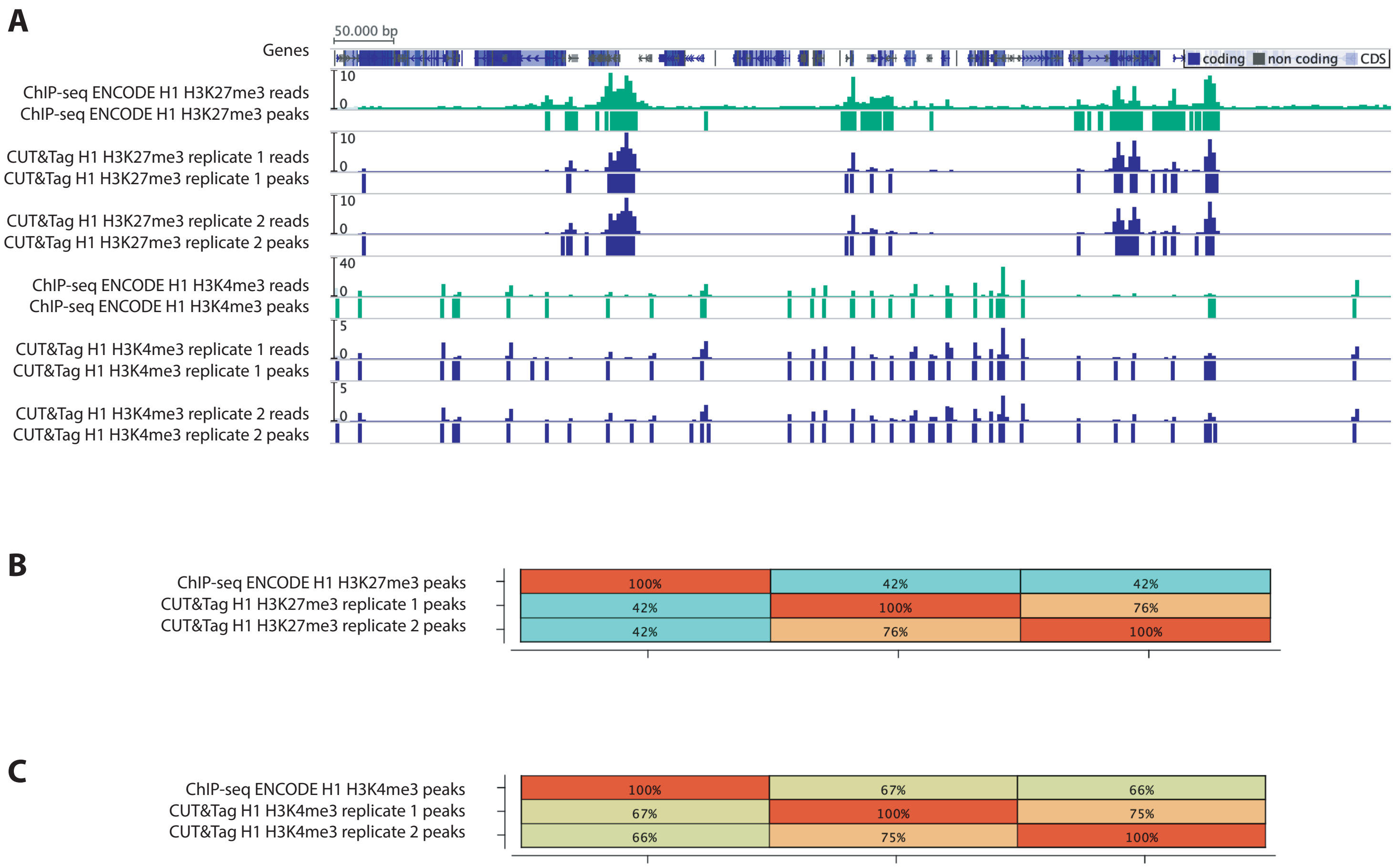

Figure S6 | CUT&Tag processing example

A, Example of peak calling results in JBR Genome Browser

B, H3K27me3 Jaccard correspondence between replicates and versus ChIP-seq data from ENCODE (JBR)

C, H3K4me3 Jaccard correspondence between replicates and versus ChIP-seq data from ENCODE (JBR)
