# Supplementary material for: Accurate chromatin marks peak calling with Omnipeak
Source: Nucleic Acids Res. 2026 Jan 9;54(1):gkaf1454. doi: 10.1093/nar/gkaf1454 (PMC12784980; doi:10.1093/nar/gkaf1454)
Supplement: gkaf1454_Supplemental_Files [file gkaf1454_supplemental_files.zip › 8_S1.pdf]

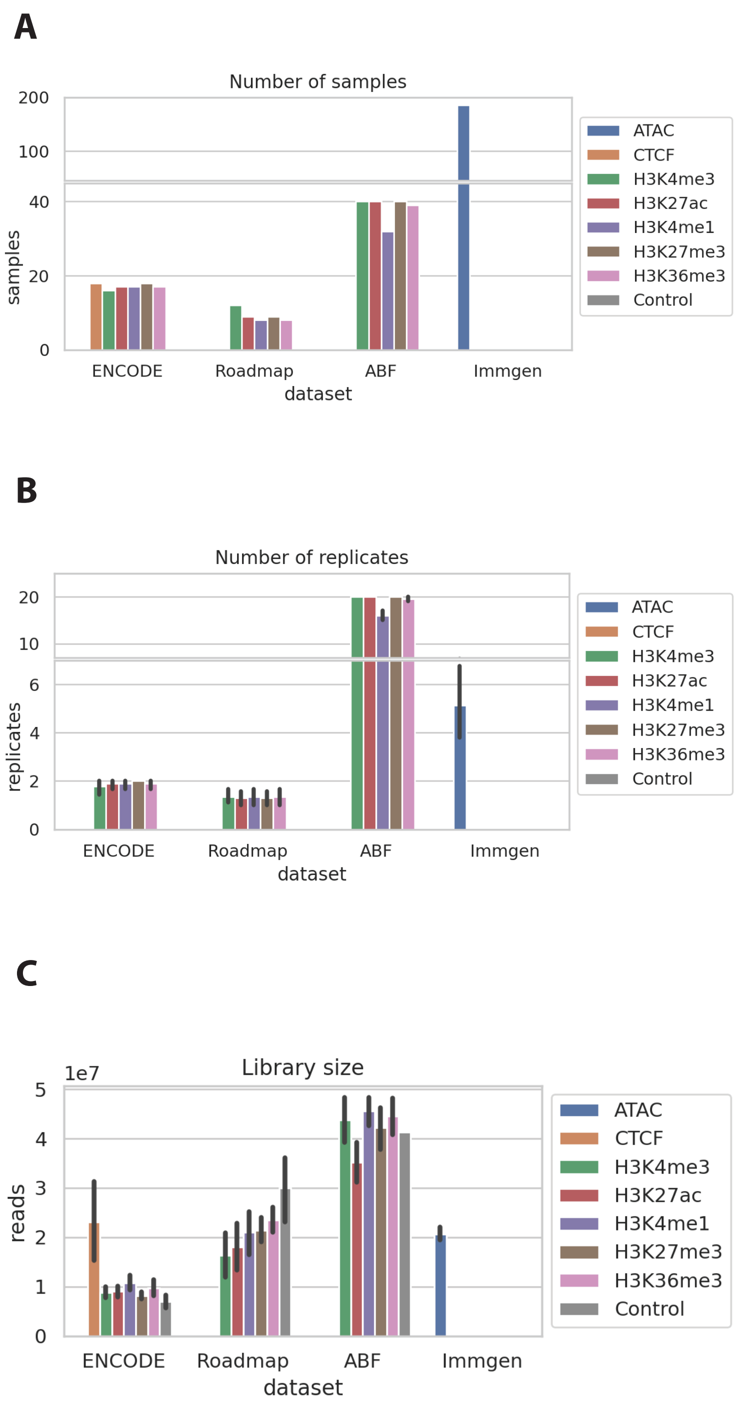

Figure S1 | Public datasets charactestics including libraries sizes and replicates numbers.
A, Total number of experiments per chromatin mark or ATAC-seq per datasets.
B, Distribution of replicates number per datasets.
C, Libraries sizes per experiment.
