# Supplementary material for: Accurate chromatin marks peak calling with Omnipeak
Source: Nucleic Acids Res. 2026 Jan 9;54(1):gkaf1454. doi: 10.1093/nar/gkaf1454 (PMC12784980; doi:10.1093/nar/gkaf1454)
Supplement: gkaf1454_Supplemental_Files [file gkaf1454_supplemental_files.zip › 9_S2.pdf]

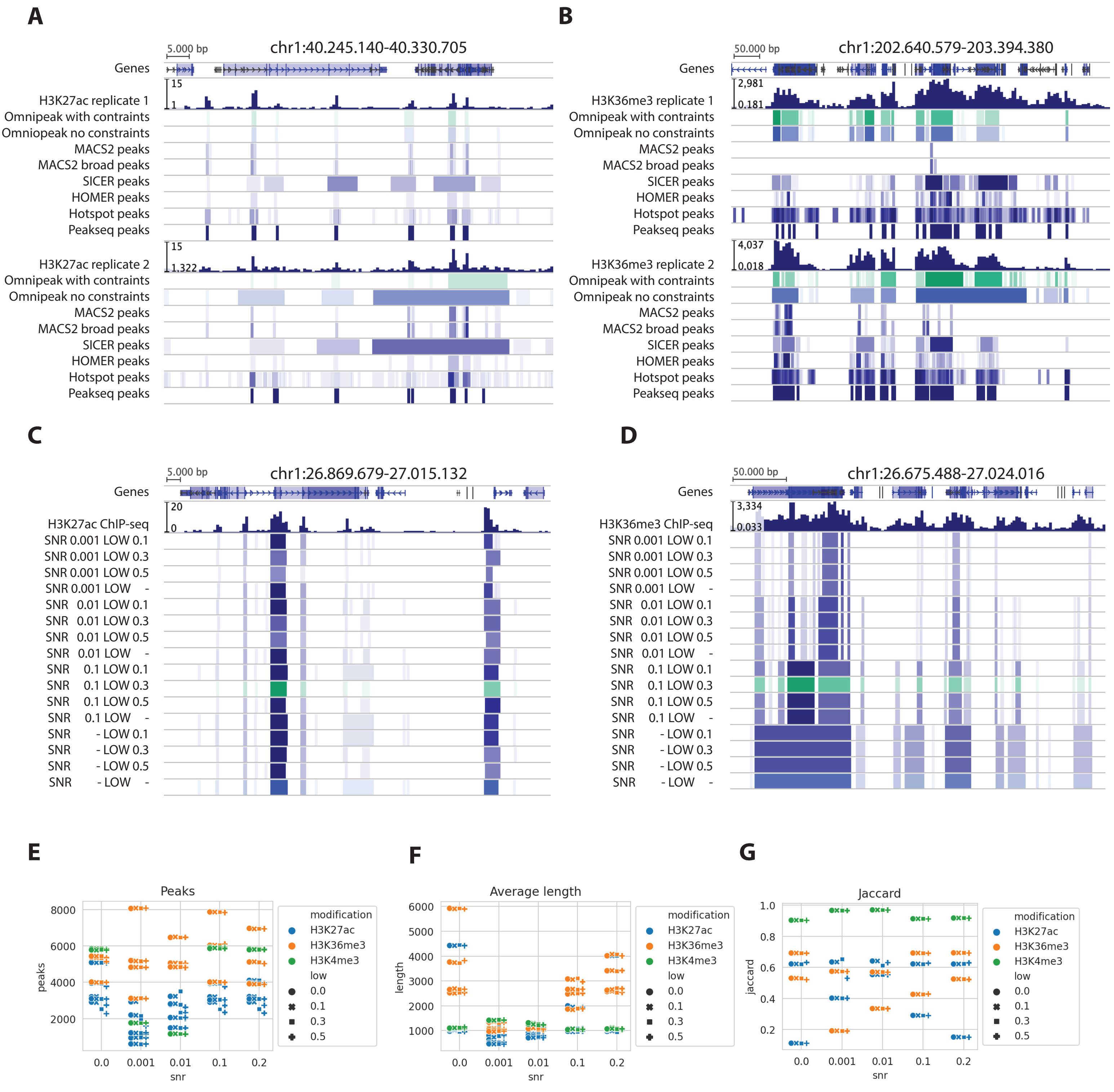

Figure S2 | Omnipeak hyperparameters influence on peak calling results.
A, Peak calling in ABF dataset of H3K27ac in two replicates (green - Omnipeak with constraints, blue - without).
B, Peak calling in ENCODE of H3K36me3 in two replicates (green - Omnipeak with constraints, blue - without).
C, Dependency of Signal-to-noise ratio (SNR) and low state (LOW) constraint on peak calling of H3K27ac mark.
D, Dependency of Signal-to-noise ratio (SNR) and low state (LOW) constraint on peak calling of H3K36me3 mark.
E, Number of peaks dependency on SNR and LOW. **F**, Average length of peaks dependency on SNR and LOW.
G, Jaccard consistency between replicates depedency on SNR and LOW.
