# Supplementary material for: Accurate chromatin marks peak calling with Omnipeak
Source: Nucleic Acids Res. 2026 Jan 9;54(1):gkaf1454. doi: 10.1093/nar/gkaf1454 (PMC12784980; doi:10.1093/nar/gkaf1454)
Supplement: gkaf1454_Supplemental_Files [file gkaf1454_supplemental_files.zip › SupplementaryFigureLegends.docx]

**SUPPLEMENTARY FIGURE LEGENDS**

Figure S1 | Public datasets charactestics including libraries sizes and replicates numbers.

1. Total number of experiments per chromatin mark or ATAC-seq per datasets.
2. Distribution of replicates number per datasets.
3. Libraries sizes per experiment.

Figure S2 | Omnipeak hyperparameters influence on peak calling results.

1. Peak calling in ABF dataset of H3K27ac in two replicates (green - Omnipeak with constraints, blue - without).
2. Peak calling in ENCODE of H3K36me3 in two replicates (green - Omnipeak with constraints, blue - without).
3. Dependency of Signal-to-noise ratio (SNR) and low state (LOW) constraint on peak calling of H3K27ac mark.
4. Dependency of Signal-to-noise ratio (SNR) and low state (LOW) constraint on peak calling of H3K36me3 mark.
5. Number of peaks dependency on SNR and LOW.
6. Average length of peaks dependency on SNR and LOW.
7. Jaccard consistency between replicates depedency on SNR and LOW.

Figure S3 | Omnipeak candidate peaks patterns look similar across marks - a key to unified procedure.

1. Candidate peaks selection procedure involves analysis of both candidates number and average candidates length, during PEP threshold variation from the most stringest to the most relaxed settings. Similar patterns present across all experiment types.
2. Similar patterns of number of candidate peaks vs various PEP theshold ranks across different experiments. In all cases there is a steady growth, saturation and optionally small decline, growth caused by yielding lots of unsignificant candidates and final merging for most relaxed PEP thresholds.
3. Candidates average length pattern is invariant across experiments, similar trajectory path as in number of peaks.
4. Visualization of novel candidates percentage while relaxing PEP threshold allows to detect saturation point.

Figure S4 | Peak calling within JBR Genome Browser with Omnipeak.

1. User starts peak calling by selecting BigWig or BAM files and choosing Peak Caller | Call peaks with OmniPeak option from the JBR Genome Browser context menu.
2. Setting for peak calling including model and peaks location selection.
3. Summary information of the generated peaks following OmniPeak execution.
4. Visualization of the resulting peaks within the JBR Genome Browser user interface.

Figure S5 | Summary rank statistics of the benchmarks (the lower the better).

1. Ranks of the average rank of Jaccard between replicates.
2. Ranks of H3K4me3 most significant peaks versus RNA-seq actively transcribed genes.
3. Ranks of the H3K36me3 Jaccard overlap with active genes.
4. Ranks of the Jaccard AUC versus ground truth peaks in simulation with Chips.
5. Ranks of of the Jaccard AUC versus peaks obtained in 100% quality simulation.
6. Ranks of the Jaccard of peaks obtained with and without control tracks.
7. Ranks of the Jaccard of peaks obtained without control tracks vs ground truth peaks in simulation.
8. Ranks of the Jaccard between replicates in ATAC-seq dataset.

Figure S6 | CUT&Tag processing example

1. Example of peak calling results in JBR Genome Browser
2. H3K27me3 Jaccard correspondence between replicates and versus ChIP-seq data from ENCODE (JBR)
3. H3K4me3 Jaccard correspondence between replicates and versus ChIP-seq data from ENCODE (JBR)
