# Supplementary material for: Accurate chromatin marks peak calling with Omnipeak
Source: Nucleic Acids Res. 2026 Jan 9;54(1):gkaf1454. doi: 10.1093/nar/gkaf1454 (PMC12784980; doi:10.1093/nar/gkaf1454)
Supplement: gkaf1454_Supplemental_Files [file gkaf1454_supplemental_files.zip › SupplementaryTableLegends.docx]

**SUPPLEMENTARY TABLE LEGENDS**

Supplementary Table 1. All the considered peak calling tools, including those not included in the comprehensive analysis.

Supplementary Table 2. The dataset information used in the analysis.

Supplementary Table 3. Genome Browser sessions for the figures.

Supplementary Table 4. Different benchmarks are available for the peak caller methods.
